# Computational Model Informs Effective Control Interventions against *Y. enterocolitica* Co-Infection

**DOI:** 10.3390/biology9120431

**Published:** 2020-11-30

**Authors:** Reihaneh Mostolizadeh, Andreas Dräger

**Affiliations:** 1 Computational Systems Biology of Infections and Antimicrobial-Resistant Pathogens, Institute for Bioinformatics and Medical Informatics (IBMI), University of Tübingen, 72076 Tübingen, Germany; draeger@informatik.uni-tuebingen.de; 2 Department of Computer Science, University of Tübingen, 72076 Tübingen, Germany; 3 German Center for Infection Research (DZIF), Partner Site Tübingen, 72076 Tübingen, Germany; 4 Cluster of Excellence ‘Controlling Microbes to Fight Infections’, University of Tübingen, 72076 Tübingen, Germany

**Keywords:** reproduction number, disease-free equilibrium, co-existence equilibrium, *Yersinia*, gastroenteritis

## Abstract

**Simple Summary:**

Medical control strategies for infectious diseases remain enormously important. One germ that can cause gastrointestinal infections is *Yersinia enterocolitica*. This study investigates and analyzes a computational model to identify the occurrence of disease-free and co-infection states. Thereby, the reproduction number $R_{0}$ informs us about the germ’s ability to spread disease. Suppose this fundamental quantity takes a value between zero and one. In that case, every infectious strain will cause less than one secondary infection, so the strain will disappear. In contrast, if $R_{0}$ exceeds one, every infectious strain causes more than one secondary infection, and *Yersinia* infection strains will persist. A disease-free state occurs when the commensal bacteria’s growth rate exceeds the maximum immune action and the rate at which the intestines release the bacteria. With a large enough commensal bacteria growth rate, this state can be stable. Co-infection occurs when the maximum growth rates of the wild-type and mutant strains become unequal. Studying the immune system’s behavior can result in an infection’s disappearance from hosts with a healthy microbiota immune system. In this case, *Yersinia* strains do not spread in the lumen when the commensal bacteria’s growth rate exceeds the growth rate of wild-type and mutant *Yersinia*.

**Abstract:**

The complex interplay between pathogens, host factors, and the integrity and composition of the endogenous microbiome determine the course and outcome of gastrointestinal infections. The model organism *Yersinia entercolitica* (Ye) is one of the five top frequent causes of bacterial gastroenteritis based on the Epidemiological Bulletin of the Robert Koch Institute (RKI), 10 September 2020. A fundamental challenge in predicting the course of an infection is to understand whether co-infection with two *Yersinia* strains, differing only in their capacity to resist killing by the host immune system, may decrease the overall virulence by competitive exclusion or increase it by acting cooperatively. Herein, we study the primary interactions among Ye, the host immune system and the microbiota, and their influence on *Yersinia* population dynamics. The employed model considers commensal bacterial in two host compartments (the intestinal mucosa the and lumen), the co-existence of wt and mut *Yersinia* strains, and the host immune responses. We determine four possible equilibria: disease-free, wt-free, mut-free, and co-existence of wt and mut in equilibrium. We also calculate the reproduction number for each strain as a threshold parameter to determine if the population may be eradicated or persist within the host. We conclude that the infection should disappear if the reproduction numbers for each strain fall below one, and the commensal bacteria growth rate exceeds the pathogen’s growth rate. These findings will help inform medical control strategies. The supplement includes the MATLAB source script, Maple workbook, and figures.

## 1. Introduction

Forecasting the evolution of infectious diseases is the primary motivation behind the use of mathematical models in biology. Determining those threshold values that predict whether the disease will spread within the host or can be contained is crucial to inform medical control strategies.

*Yersinia entercolitica* (Ye) is a Gram-negative enteropathogen causing foodborne gastrointestinal infections. Within the small intestine (SI), Ye can adhere to and invade the intestinal epithelial lining mainly via the adhesins Yersinia adhesin A (YadA) [1] and Invasin [2,3,4]. YadA is among the essential virulence factors as a YadA-deficient strain is impaired 97 colonizations, and systemic spread in a mouse model of infection has been shown Upon attachment, Ye can engage its Type III secretion system (T3SS) to inject effector proteins, the *Yersinia* outer proteins (Yops), into host cells, thus evading the host immune response and establishing a productive infection [5,6,7]. Indeed, the first line of host defense against invading Ye is a massive infiltration of phagocytotic cells. However, Ye can counteract phagocytic killing via its T3SS [6,7]. Together, both the T3SS and YadA contribute to the efficient colonization of the intestinal tract, where Ye induces an inflammatory response that likely accounts for a reduction in density and complexity of the commensal microbiome [8,9]. Several Ye serotypes have been isolated from animal reservoirs and the human gastrointestinal tract, but only a few of them cause disease in humans. Although causing severe distress, gastrointestinal infections are generally self-contained. Typically, healthy persons will only receive symptomatic treatment aimed at avoiding dehydration. However, in individuals with underlying disease, elderly persons, or newborn children, gastrointestinal infections can cause high morbidity and even fatal outcomes. Especially in such patients, identifying those at high risk of developing fatal diseases could help tailor personalized therapeutic interventions to improve the outcome of infection. To this aim, we recently developed a computational model that can calculate Ye population dynamics during gastrointestinal infection and predict pathogen expansion, gut colonization, and infection course [10].

Previously published models have focused on the virulence evolution during co-infection and superinfection by more than one pathogen [11,12,13,14,15,16,17]. Furthermore, Nurtay et al. [18] investigated theoretical conditions in co-existence strains in their research focused on viral populations of wt and mut [18]. Herein, we apply our computational model to predict the behavior of Ye population dynamics during the co-infection of mice by different Ye mutant (mut) strains, lacking distinct virulence factors, and a Ye wt strain in bacterial gastroenteritis. We perform a bifurcation analysis to show how the dynamics of the system change as multiple parameters are varied [19,20].

The existence of a backward bifurcation, i.e., the co-existence of stable disease-free equilibrium with one or more stable endemic equilibria, has significant consequences on the process of producing medical policies aimed at eradicating or controlling an infectious disease within the host [21]. A fundamental parameter in models displaying a backward bifurcation is the primary reproduction number $R_{0}$. $R_{0}$ represents the expected number of new infections caused within the host or between hosts when a pathogen enters into the host. If $R_{0}>1$, after an initial introduction, the infection spreads within the host/or between hosts, thus creating an infectious disease. If $R_{0}<1$, small initial introductions are not sufficiently transmissible to spread the infection within the host (or between hosts), and the cells get infected within a host. This is called an endemic disease that will fade out. Thus, many control policies like medication/vaccination have focused on reaching coverage levels sufficient to reduce $R_{0}$ below 1 [15,16,22,23]. Therefore, in this analysis, we compute the system’s equilibrium points, study their stability and the center manifold, and investigate how the different points would affect the infection rate, virulence, and mutation rate. Next, we calculate the primary reproductive number for scenarios in which multiple strains are introduced, and an $R_{0}$ number is calculated for each strain. Finally, we discuss the biological implications of our results.

## 2. Materials and Methods

### 2.1. Model Description

**Figure 1:**
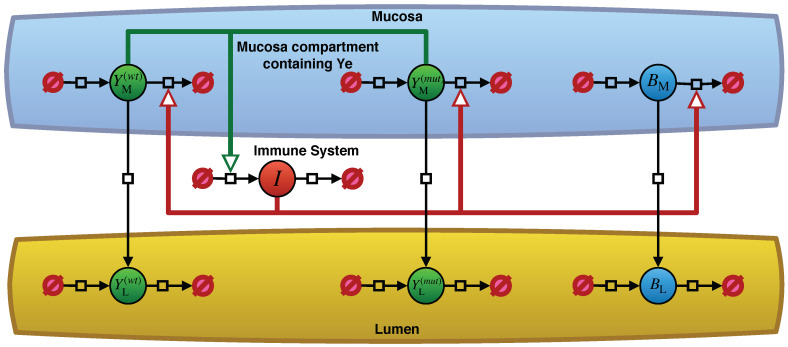
An overview of the *Yersinia enterocolitica* population dynamics model. Filled circles represent the entity pool nodes for the populations of Ye in their respective compartments as well as the strength of the immune reaction *I*. Table 1 explains the notations used in the model and this figure. The black arrows represent processes with an impact on the population dynamics of the entity pools. Arrows pointing from empty set symbols to pool nodes denote an increase in the population size or a decrease if the process arcs point from entity pools to empty sets. Migration across compartments of the respective populations appears as vertical process arcs. Some of these processes receive stimulating effects from the immune reaction or from the size of the Ye populations within the mucosa, as colored arcs indicate. Reference [10] provides a more detailed description of the model’s structure.

Our model for Ye, as described in Geißert et al. [10], considers three different sites with their individual population dynamics. Figure 1 depicts an overview of the model in the form of a Systems Biology Graphical Notation (SBGN) [24] PD diagram [25] based on an illustration by Geißert et al. [10]. The lumen and the mucosal site of the small intestine are illustrated as two separate compartments by abbreviated notations *L* and *M* in Table 1. The mucosa and lumen sites include three different population dynamics: commensal bacteria, wt *Yersinia*, and mut *Yersinia*.

Besides the bacterial populations, we have the strength of the host immune response. For the sake of clarity, the complex immune cell population dynamics, which is made up of innate and adaptive immunity, are implemented in our model as a single abstract immune response in Table 1. An advantage of this rather abstract immune response is that it is easily adjustable. Only the Ye populations in the mucosa activate the immune response, whereas tolerance to commensal bacteria exists. However, this immune response affects all bacterial populations at the mucosal site. Upon oral co-infection, a Ye population enters the small intestinal lumen. Due to their particular virulence traits, some Ye cells are also able to enter the mucosal site.

As shown in Models (1)–(7), the population dynamics of bacterial species consist of both growth, i.e., an increase in populations due to a distinct growth rate α, and a reduction through peristalsis, which moves the bacterial populations towards the colon where they finally end up in feces. Peristalsis will only influence the populations in the lumen. Furthermore, bacterial populations in the mucosa can additionally be reduced through killing by the host immune attack.

Both compartments (the lumen and the mucosal) are colonized by commensal bacterial species but to different extents. The colonization capacity of the lumen considerably exceeds that of the mucosal site. This is due to the host’s natural mechanisms to limit bacterial contact to the epithelium through physical barriers, such as the adherent mucus layer, and a high concentration of anti-microbial peptides (AMPs). Hence, we assume a much lower carrying capacity of the mucosal site compared to the luminal compartment. Another assumption is that as soon as bacterial numbers exceed the mucosa’s carrying capacity, they spill-over to the luminal site, feeding the luminal populations [10].

The model’s immune system represented by *I* (see Table 1) is activated as soon as at least one Ye cell enters the small intestinal mucosal compartment. This immune activation increases with the number of Ye cells in the mucosa. By triggering the killing of all bacterial cells at the mucosal site, this leads to a reduction in bacterial populations spilling over to the lumen [10].

In contrast to commensal bacteria, Ye exhibits a number of virulence traits to evade the host immune response. This is implemented in our model by different immunity adjustment factors for either the wt strain or the different mut strains. Consequently, the number of commensal species is more affected by the host immune attack than the number of Ye mut strains, which are, of course, more affected than the wt strain. Our model’s final output is the number of bacteria, or colony-forming units (CFUs), finally ending up in feces. These population dynamics are represented as the following system of differential equations [10]:

(1) $$\begin{array}{cc} \frac{d}{dt}B_{M} & =\left(\alpha^{\left(B\right)}-\sigma_{M⟶L}^{\left(B\right)}-\gamma \;\cdot \;I\right)\;\cdot \;B_{M} \end{array}$$

(2) $$\begin{array}{cc} \frac{d}{dt}Y_{M}^{\left(wt\right)} & =\left(\alpha^{\left(wt\right)}-\sigma_{M⟶L}^{\left(wt\right)}-\gamma \;\cdot \;f_{I}^{\left(wt\right)}\;\cdot \;I\right)\;\cdot \;Y_{M}^{\left(wt\right)} \end{array}$$

(3) $$\begin{array}{cc} \frac{d}{dt}Y_{M}^{\left(mut\right)} & =\left(\alpha^{\left(mut\right)}-\sigma_{M⟶L}^{\left(mut\right)}-\gamma \;\cdot \;f_{I}^{\left(mut\right)}\;\cdot \;I\right)\;\cdot \;Y_{M}^{\left(mut\right)} \end{array}$$

(4) $$\begin{array}{cc} \frac{d}{dt}B_{L} & =\left(\alpha_{L}^{\left(B\right)}-\beta \right)\;\cdot \;B_{L}+\sigma_{M⟶L}^{\left(B\right)}\;\cdot \;B_{M} \end{array}$$

(5) $$\begin{array}{cc} \frac{d}{dt}Y_{L}^{\left(wt\right)} & =\left(\alpha_{L}^{\left(wt\right)}-\beta \right)\;\cdot \;Y_{L}^{\left(wt\right)}+\sigma_{M⟶L}^{\left(wt\right)}\;\cdot \;Y_{M}^{\left(wt\right)} \end{array}$$

(6) $$\begin{array}{cc} \frac{d}{dt}Y_{L}^{\left(mut\right)} & =\left(\alpha_{L}^{\left(mut\right)}-\beta \right)\;\cdot \;Y_{L}^{\left(mut\right)}+\sigma_{M⟶L}^{\left(mut\right)}\;\cdot \;Y_{M}^{\left(mut\right)} \end{array}$$

(7) $$\begin{array}{cc} \frac{d}{dt}I & =\left(Y_{M}^{\left(wt\right)}+Y_{M}^{\left(mut\right)}\right)\;\cdot \;\kappa \;\cdot \;\frac{C_{I}-I}{C_{I}}. \end{array}$$

For a detailed description of the parameters, see Table 2. Besides the seven differential equations describing the population dynamics of the Ye strains, the commensal bacteria and the immune system at the mucosal site, and all three populations (commensal bacteria, wt *Yersinia*, and mut *Yersinia*) at the luminal site, the model consists of different mass-action terms describing the spill-over rates from the mucosa into the lumen and describes the growth rates of the bacterial populations, which are limited through a given capacity “C” and are defined as follows:

(8) $$\begin{array}{ccc} \sigma_{M⟶L}^{\left(B\right)} & =\alpha^{\left(B\right)}\frac{B_{M}+Y_{M}^{\left(wt\right)}+Y_{M}^{\left(mut\right)}}{C_{M}} & \text{Spill-over}\;\mathrm{rate}\;\mathrm{commensal} \end{array}$$

(9) $$\begin{array}{ccc} \sigma_{M⟶L}^{\left(wt\right)} & =\alpha^{\left(wt\right)}\frac{B_{M}+Y_{M}^{\left(wt\right)}+Y_{M}^{\left(mut\right)}}{C_{M}} & \text{Spill-over}\;\mathrm{rate}\;\mathrm{wt} \end{array}$$

(10) $$\begin{array}{ccc} \sigma_{M⟶L}^{\left(mut\right)} & =\alpha^{\left(mut\right)}\frac{B_{M}+Y_{M}^{\left(wt\right)}+Y_{M}^{\left(mut\right)}}{C_{M}} & \text{Spill-over}\;\mathrm{rate}\;\mathrm{mut} \end{array}$$

(11) $$\begin{array}{ccc} \alpha_{L}^{\left(B\right)} & =\alpha^{\left(B\right)}\frac{C_{L}-\left(B_{L}+Y_{L}^{\left(wt\right)}+Y_{L}^{\left(mut\right)}\right)}{C_{L}} & \mathrm{Bacteria}\;\mathrm{growth}\;\mathrm{rate}\;\mathrm{in}\;\mathrm{the}\;\mathrm{lumen} \end{array}$$

(12) $$\begin{array}{ccc} \alpha_{L}^{\left(wt\right)} & =\alpha^{\left(wt\right)}\frac{C_{L}-\left(B_{L}+Y_{L}^{\left(wt\right)}+Y_{L}^{\left(mut\right)}\right)}{C_{L}} & \mathrm{wt}\;\mathrm{growth}\;\mathrm{rate}\;\mathrm{in}\;\mathrm{the}\;\mathrm{lumen} \end{array}$$

(13) $$\begin{array}{ccc} \alpha_{L}^{\left(mut\right)} & =\alpha^{\left(mut\right)}\frac{C_{L}-\left(B_{L}+Y_{L}^{\left(wt\right)}+Y_{L}^{\left(mut\right)}\right)}{C_{L}} & \mathrm{mut}\;\mathrm{growth}\;\mathrm{rate}\;\mathrm{in}\;\mathrm{the}\;\mathrm{lumen} \end{array}$$

Model parameters are shown in Table 2. Since the system models the *Yersinia* population in the mucosa and lumen, we assume that all state variables and parameters of the model are non-negative ∀t≥0.

### 2.2. The Basic Reproduction Number $R_{0}$

The basic reproduction ratio $R_{0}$ is calculated by the fraction of the transmission rate for each strain (spill-over) and the average infectious period for each strain in compartments, as defined by Diekmann et al. [28].

In addition, van den Driessche and Watmough [29,30] defined $R_{0}$ as a general compartmental disease transmission model suited to heterogeneous populations that can be modeled by a system of ordinary differential equations. Suppose there are *n* disease compartments and *m* non-disease compartments, and let $x\in R^{n}$ and $y\in R^{m}$ be the subpopulations in each of these compartments. In this splitting, the authors thus represented the diseased compartment with $F_{i}\left(x\right)$ as the rate of the appearance of new infections in compartment *i*, and $V_{i}$ as the rate disease progression, death, and recovery reduced in compartment *i*, which can be written as ${\dot{x}}_{i}=f_{i}\left(x\right)=F_{i}\left(x\right)-V_{i},\;\;\;i=1,\ldots ,n$.

To understand compartment $V_{i}$ better, this can be written as $V_{i}=V_{i}^{-}+V_{i}^{-}$, where $V_{i}^{+}$ is defined as the rate of transfer of individuals into compartment *i* by all other means, and $V_{i}^{-}$ is the rate of transfer of individuals out of the compartment *i*. Therefore, the disease transmission model consisting of non-negative initial conditions will be as follows:

(14) $$\begin{array}{cccc} \frac{d}{dt}x_{i} & =F_{i}\left(x,y\right)-V_{i}\left(x,y\right) & \forall i & \in \{1,\ldots ,n\} \end{array}$$

(15) $$\begin{array}{cccc} \frac{d}{dt}y_{j} & =G_{j}\left(x,y\right) & \forall j & \in \{1,\ldots ,m\}. \end{array}$$

It is assumed that functions $F_{i}\left(x\right)$ and $V_{i}=V_{i}^{-}+V_{i}^{-}$ are continuously differentiable at least twice in each variable, and they satisfy the assumptions A(1)–A(5) described below [29,31]:

A(1) $F_{i}\left(0,y\right)=0,\;V_{i}\left(0,y\right)=0:\forall y>0\;\;\;\mathrm{and}\;\;\;i=1,\ldots ,n$ (no immigration of individuals into the disease compartments)
A(2) $F_{i}\left(x,y\right)\geq 0:\forall x_{i}\geq 0\wedge y_{i}\geq 0\;\;\;\mathrm{and}\;\;\;i=1,\;\;\;\ldots ,n$ (the new infections will be represented by F, so it cannot be negative)
A(3) $V_{i}\left(x,y\right)\leq 0:\;\mathrm{whenever}\;\;\;x_{i}=0,\;\;\;i=1,\ldots ,n$ (if the compartment is empty, it can only have inflow, and the net outflow from the compartment must be negative)
A(4) $\sum_{i}V_{i}\left(x,y\right)\geq 0:\forall x_{i}\geq 0\wedge y_{i}\geq 0$ (sum is net outflow)
A(5) The system $\dot{y}=G\left(0,y\right)$ has a unique asymptotically stable equilibrium, $y^{*}$ (all solutions with initial conditions of the form (0,y) approach a point $(0,y^{*})\;\;\mathrm{as}\;\;t\to \infty$)

**Theorem** **1.** *Consider the disease transmission model given by* $\dot{X}=f\left(X\right)$ *with* f(X) *satisfying conditions A(1)–A(5). If* $X_{0}$ *is a DFE of the model, then* $X_{0}$ *is locally asymptotically stable if* $R_{0}<1$ *, but unstable if* $R_{0}>1$ *, where* $R_{0}$ *acts as a threshold parameter.*

**Proof.**  See [29]. □

By defining $F=\frac{\partial F_{i}}{\partial x_{j}}\left(0,y^{*}\right)$ and $V=\frac{\partial V_{i}}{\partial x_{j}}\left(0,y^{*}\right)$ as n×n matrices, the basic reproduction number $R_{0}$ is computed by $R_{0}=\rho \left(FV^{-1}\right)$, where ρ(A) denotes the spectral radius of a matrix *A*.

Several models found in the literature have been used to show that when $R_{0}$ crosses the threshold, $R_{0}=1$, it can act as a bifurcation parameter and transcritical bifurcation takes place. That is, local asymptotical stability is transferred from the disease-free state to the new (positive) equilibria. In ordinary differential equations, we will encounter bifurcations of equilibrium and periodic orbits, which are typical in the sense that they occur when a small smooth change is made to the threshold parameter values (the bifurcation parameters μ) of a system. It causes a sudden qualitative or topological change in the behavior of the system. Consider the continuous dynamical system described by the Ordinary Differential Equation (ODE)

(16) $$\dot{X}=f\left(X,\mu \right)\;\mathrm{with}\;f:R^{n}\times R\to R^{n}.$$

A local bifurcation occurs at $\left(X_{0},\mu_{0}\right)$ if an eigenvalue with zero real part is included in the Jacobian matrix of the system. If the eigenvalue is equal to zero, the bifurcation is a steady-state bifurcation. Therefore, we now recall the analysis of the center manifold near the critical $\left(X=X_{0},R_{0}=1\right)$, which allows clarifying the direction of the bifurcation near the bifurcation point using the Center Manifold Theorem 2. This theory describes the local stability at the non-hyperbolic equilibrium (linearization matrix has at least one eigenvalue with zero real parts) and the existence of another equilibrium (bifurcated from the non-hyperbolic equilibrium).

### 2.3. Center Manifold

The center manifold theorem provides a means for systematically reducing the dimensions of the state spaces, which need to be considered when analyzing bifurcations of a given type.

**Theorem** **2** (Center Manifold Theorem for Flows)**.** *Let f be a* $C^{r}$ *vector field on* $R^{n}$ *vanishing at the origin* f(0)=0 *, and let* A=Df(0) *. Divide the spectrum of A into three parts,* $\sigma_{s},\sigma_{c},and\sigma_{u}$ *, with*

(17) $$Re\lambda \left\{\begin{array}{cc} <0 & \mathrm{if}\;\lambda \in \sigma_{s}\\ =0 & \mathrm{if}\;\lambda \in \sigma_{c}\\ >0 & \mathrm{if}\;\lambda \in \sigma_{u} \end{array}\right..$$

*Let the (generalized) eigenspaces of*$\sigma_{s},\sigma_{c}$*, and*$\sigma_{u}$*be*$E^{s},E^{c}$*, and*$E^{u}$*, respectively. Then, there exists*$C^{r}$*stable and unstable invariant manifolds*$W^{u}$*and*$W^{s}$*tangent to*$E^{u}$*and*$E^{s}$*at* 0 *and a* $C^{r-1}$ *center manifold* $W^{c}$ *tangent to* $E^{c}$ *at* 0*. The manifolds* $W^{u},W^{s}$*, and* $W^{c}$ *are all invariant for the flow of f. Stable and unstable manifolds are unique, but* $W^{c}$ *need not be.*

**Proof.**  See [32,33]. □

To achieve this, consider the general system

(18) $$\begin{array}{c} \dot{x}=Jx+F\left(x\right),\;x\in R^{n} \end{array}$$

where Jx is the linear part of the system. We must first find the differential equations on its center manifold and then reduce the system to its normal form. Without loss of generality, we assume that x=0 is the fixed point of interest for the system.

Suppose *J* has $n_{c}$ eigenvalues with zero real-parts and $n_{s}$ eigenvalues with negative and positive real parts, and $n=n_{c}+n_{s}$. Using the eigenvectors of *J* to form a transformation matrix, the system can be rewritten in block matrix form as

(19) $$\left\{\begin{array}{ccc} {\dot{x}}_{c} & = & Ax_{c}+f\left(x_{c},x_{s}\right)\\ {\dot{x}}_{s} & = & Bx_{s}+g\left(x_{c},x_{s}\right) \end{array}\right.$$

where $\left(x_{c},x_{s}\right)\in R^{n_{c}}\times R^{n_{s}}$, $A\in R^{n_{c}}\times R^{n_{c}}$, and $B\in R^{n_{s}}\times R^{n_{s}}$. With the eigenvalues of zero real parts, the Center Manifold Theorem 2 guarantees that there exists a smooth manifold $W_{c}=\left\{\left(x_{c},x_{s}\right)|x_{s}=q\left(x_{c}\right)\right\}$, the equilibrium point such that the local behavior in the center direction of the system is qualitatively the same as that on the manifold. By differentiating $x_{s}=q\left(x_{c}\right)$, we get ${\dot{x}}_{s}=Dq\left(x_{c}\right){\dot{x}}_{c}$. Substituting (19) into the previous identity and rearranging the equation, we get

(20) $$Dq\left(x_{c}\right)\left[Ax_{c}+f\left(x_{c},q\left(x_{c}\right)\right)\right]-Bq\left(x_{c}\right)-g\left(x_{c},q\left(x_{c}\right)\right)=0.$$

By solving for $q(x_{c})$, we get a function describing the center manifold. In general, $q(x_{c})$ cannot be solved explicitly. Instead, substituting a Taylor expansion $q\left(x_{c}\right)=ax_{c}^{2}+O\left(x_{c}^{3}\right)$ into (20), we can find the coefficients for the expansion by balancing the lower-order terms. Based on $q(x_{c})$, we now have a system in the reduced form:

(21) $${\dot{x}}_{c}=Ax_{c}+f\left(x_{c},q\left(x_{c}\right)\right).$$

The following proposition helps us understand if the type of transcritical bifurcation is forward or backward.

**Proposition** **1.** *Assume that conditions A(1)–A(5) are satisfied. Furthermore, assume that the following hypotheses are satisfied by the system in (14) and (15):*

*H(1)*  *In the balance equations for the infected compartments, nonlinear terms are present only in the rate of the appearance of new infections;*
*H(2)*  *Nonlinear terms are bilinear;*
*H(3)*  *There is no linear transfer from infected to uninfected compartments.*

*Then, the transcritical bifurcation of the system in (14) and (15) at* $R_{0}=1$ *is forward, and if at least one of these features is not present in the model structure, then a backward bifurcation may occur [31].*

## 3. Results

We used the model in (1)–(7) to understand how the dynamics change following variations of different parameters. We calculated the equilibria of (1)–(7), conducted a linear stability analysis, and identified the analytical conditions that lead to a transcritical bifurcation.

### 3.1. Existence of Equilibria

For mathematical convenience, we divide the model in (1)–(7) such that the first four equations correspond to infected individuals. The distinction between infected and uninfected populations must be determined from the model’s epidemiological interpretation and cannot be deduced from the structure of the equations alone. Thus, we have

(22) $$\begin{array}{cc} \frac{d}{dt}Y_{M}^{\left(wt\right)} & =\left(\alpha^{\left(wt\right)}-\sigma_{M⟶L}^{\left(wt\right)}-\gamma \;\cdot \;f_{I}^{\left(wt\right)}\;\cdot \;I\right)\;\cdot \;Y_{M}^{\left(wt\right)} \end{array}$$

(23) $$\begin{array}{cc} \frac{d}{dt}Y_{M}^{\left(mut\right)} & =\left(\alpha^{\left(mut\right)}-\sigma_{M⟶L}^{\left(mut\right)}-\gamma \;\cdot \;f_{I}^{\left(mut\right)}\;\cdot \;I\right)\;\cdot \;Y_{M}^{\left(mut\right)} \end{array}$$

(24) $$\begin{array}{cc} \frac{d}{dt}Y_{L}^{\left(wt\right)} & =\left(\alpha_{L}^{\left(wt\right)}-\beta \right)\;\cdot \;Y_{L}^{\left(wt\right)}+\sigma_{M⟶L}^{\left(wt\right)}\;\cdot \;Y_{M}^{\left(wt\right)} \end{array}$$

(25) $$\begin{array}{cc} \frac{d}{dt}Y_{L}^{\left(mut\right)} & =\left(\alpha_{L}^{\left(mut\right)}-\beta \right)\;\cdot \;Y_{L}^{\left(mut\right)}+\sigma_{M⟶L}^{\left(mut\right)}\;\cdot \;Y_{M}^{\left(mut\right)} \end{array}$$

(26) $$\begin{array}{cc} \frac{d}{dt}B_{M} & =\left(\alpha^{\left(B\right)}-\sigma_{M⟶L}^{\left(B\right)}-\gamma \;\cdot \;I\right)\;\cdot \;B_{M} \end{array}$$

(27) $$\begin{array}{cc} \frac{d}{dt}B_{L} & =\left(\alpha_{L}^{\left(B\right)}-\beta \right)\;\cdot \;B_{L}+\sigma_{M⟶L}^{\left(B\right)}\;\cdot \;B_{M} \end{array}$$

(28) $$\begin{array}{cc} \frac{d}{dt}I & =\left(Y_{M}^{\left(wt\right)}+Y_{M}^{\left(mut\right)}\right)\;\cdot \;\kappa \;\cdot \;\frac{C_{I}-I}{C_{I}}, \end{array}$$

while we assume that the growth rate $\alpha^{\left(B\right)}$ of the endogenous commensal bacteria is higher than the Ye growth rates $\alpha^{\left(wt\right)}$ and $\alpha^{\left(mut\right)}$, respectively.

On the other side, the *Yersinia* model has three compartments (mucosa, lumen, immune system), which are analyzed separately. The model system is analyzed in a suitable, feasible region. All forward solutions of the system are feasible ∀t≥0 if they enter the invariant region $\Omega =\Omega_{L}\times \Omega_{M}\times \Omega_{I}$ where

(29) $$\begin{array}{cc} \Omega_{L} & =\left(B_{L},Y_{L}^{\left(wt\right)},Y_{L}^{\left(mut\right)}\right)\in R_{+}^{3}:B_{L}+Y_{L}^{\left(wt\right)}+Y_{L}^{\left(mut\right)}\leq C_{L} \end{array}$$

(30) $$\begin{array}{cc} \Omega_{M} & =\left(B_{M},Y_{M}^{\left(wt\right)},Y_{M}^{\left(mut\right)}\right)\in R_{+}^{3}:B_{M}+Y_{M}^{\left(wt\right)}+Y_{M}^{\left(mut\right)}\leq C_{M} \end{array}$$

(31) $$\begin{array}{cc} \Omega_{I} & =\left(I\right)\in R_{+}^{1}:I\leq C_{I}. \end{array}$$

The existence of equilibrium points for system (1)–(7) would be as follows:

- The trivial equilibrium point is as an origin equilibrium $\left(0,0,0,0,0,0,0\right)$. This solution appears when all populations are extinct. For all parameters, this point never becomes stable due to the positivity of eigenvalues in (A2).
- The first equilibrium point appears in the absence of *Yersinia* $Y_{M}^{\left(wt\right)}=Y_{M}^{\left(mut\right)}=Y_{L}^{\left(wt\right)}=Y_{L}^{\left(mut\right)}=0$. System (1)–(7) has a disease-free equilibrium, which is given by
  (32) $$P^{0}=\left(B_{M}^{0},Y_{M}^{0(wt)},Y_{M}^{0(mut)},B_{L}^{0},Y_{L}^{0(wt)},Y_{L}^{0(mut)},I^{0}\right)=\left(B_{M}^{0},0,0,B_{L}^{0},0,0,I^{0}\right)$$
  and
  (33) $$\begin{array}{cc} B_{M}^{0} & =C_{M}\left(1-\frac{\gamma }{\alpha^{\left(B\right)}}\right) \end{array}$$
  (34) $$\begin{array}{cc} B_{L}^{0} & =C_{L}\left(1-\frac{\beta }{\alpha^{\left(B\right)}}\right) \end{array}$$
  (35) $$\begin{array}{cc} I^{0} & =1. \end{array}$$
  It describes a disease-free state whereby only the commensal bacteria persist. In order for the disease-free state $P^{0}$ to be biologically meaningful, the conditions $\gamma <\alpha^{\left(B\right)}$ and $\beta <\alpha^{\left(B\right)}$ must hold. These conditions correspond to the maximal growth rate of intestinal bacteria exceeding the rate at which intestines are charged and the maximal immunity action, which is not that strong in the absence of *Yersinia* strains. However, the population of the immune system is at its maximum carrying capacity (in health, not in fighting with any infection).
- A second equilibrium corresponds to the commensal bacteria’s persistence and the *Yersinia* mut strain in the absence of the wt strain. Without loss of generality, the commensal bacteria are supposed to be zero because they are not infective. This point is obtained by setting $Y_{M}^{\left(wt\right)}=Y_{L}^{\left(wt\right)}=0$:
  (36) $$\begin{array}{c} P^{\left(mut\right)}=P^{1}=\left(B_{M}^{1},Y_{M}^{1(wt)},Y_{M}^{1(mut)},B_{L}^{1},Y_{L}^{1(wt)},Y_{L}^{1(mut)},I^{1}\right)\\ =\left(0,0,Y_{M}^{1(mut)},0,0,Y_{L}^{1(mut)},C_{I}\right) \end{array}$$
  with
  (37) $$\begin{array}{cc} Y_{M}^{1(mut)} & =C_{M}\left(1-\frac{C_{I}\gamma f_{I}^{\left(mut\right)}}{\alpha^{\left(mut\right)}}\right) \end{array}$$
  (38) $$\begin{array}{cc} Y_{L}^{1(mut)} & =\frac{1}{2}\left(C_{L}\left(1-\frac{\beta }{\alpha^{\left(mut\right)}}\right)+\sqrt{4C_{M}C_{L}{\left(\frac{C_{I}\gamma f_{I}^{\left(mut\right)}}{\alpha^{\left(mut\right)}}-1\right)}^{2}+C_{L}^{2}{\left(1-\frac{\beta }{\alpha^{\left(mut\right)}}\right)}^{2}}\right). \end{array}$$
- The other equilibrium corresponds to the persistence of commensal bacteria and the *Yersinia* wt strain in the absence of the mut strain. Without loss of generality, the commensal bacteria are supposed to be zero because they are not infective. This point is obtained by setting $Y_{M}^{\left(mut\right)}=Y_{L}^{\left(mut\right)}=0$:
  (39) $$\begin{array}{c} P^{\left(wt\right)}=P^{2}=\left(B_{M}^{2},Y_{M}^{2(wt)},Y_{M}^{2(mut)},B_{L}^{2},Y_{L}^{2(wt)},Y_{L}^{2(mut)},I^{2}\right)=\left(0,Y_{M}^{2(wt)},0,0,Y_{L}^{2(wt)},0,C_{I}\right) \end{array}$$
  with
  (40) $$\begin{array}{cc} Y_{M}^{2(wt)} & =C_{M}\left(1-\frac{C_{I}\gamma f_{I}^{\left(wt\right)}}{\alpha^{\left(wt\right)}}\right) \end{array}$$
  (41) $$\begin{array}{cc} Y_{L}^{2(wt)} & =\frac{1}{2}\left(C_{L}\left(1-\frac{\beta }{\alpha^{\left(wt\right)}}\right)+\sqrt{4C_{M}C_{L}{\left(\frac{C_{I}\gamma f_{I}^{\left(wt\right)}}{\alpha^{\left(wt\right)}}-1\right)}^{2}+C_{L}^{2}{\left(1-\frac{\beta }{\alpha^{\left(wt\right)}}\right)}^{2}}\right). \end{array}$$
- Finally, the last equilibrium point corresponds to a state of the co-existence of wt and mut *Yersinia* strains. This point is achieved by supposing $B_{M}=B_{L}=0$:
  (42) $$\begin{array}{c} P^{\left(wt)(mut\right)}=P^{3}=\left(B_{M}^{3},Y_{M}^{3(wt)},Y_{M}^{3(mut)},B_{L}^{3},Y_{L}^{3(wt)},Y_{L}^{3(mut)},I^{3}\right)\\ =\left(0,Y_{M}^{3(wt)},Z,0,Y_{L}^{3(wt)},Y_{L}^{3(mut)},C_{I}\right) \end{array}$$
  with
  (43) $$\begin{array}{cc} Y_{M}^{3(wt)} & =C_{M}\left(1-\frac{C_{I}\gamma f_{I}^{\left(wt\right)}}{\alpha^{\left(wt\right)}}\right)-Z \end{array}$$
  (44) $$\begin{array}{cc} Y_{L}^{3(wt)} & =\frac{\alpha^{\left(mut\right)}\left(Z\left(1-\frac{C_{I}\gamma f_{I}^{\left(wt\right)}}{\alpha^{\left(wt\right)}}\right)-\gamma \;f_{I}^{\left(wt\right)}{\left(1-\frac{C_{I}}{\alpha^{\left(wt\right)}}\right)}^{2}\right)}{\beta \left(1-\frac{\alpha^{\left(mut\right)}}{\alpha^{\left(wt\right)}}\right)} \end{array}$$
  (45) $$\begin{array}{cc} Y_{L}^{3(mut)} & =\frac{\alpha^{\left(mut\right)}\left(1-\frac{C_{I}\gamma f_{I}^{\left(wt\right)}}{\alpha^{\left(wt\right)}}\right)\left(Z-\left(1-\frac{C_{I}\gamma f_{I}^{\left(wt\right)}}{\alpha^{\left(wt\right)}}\right)\right)}{\beta \left(1-\frac{\alpha^{\left(mut\right)}}{\alpha^{\left(wt\right)}}\right)} \end{array}$$
  where *Z* is defined as $\left(1-\frac{\alpha^{\left(B\right)}f_{I}^{\left(wt\right)}}{\alpha^{\left(wt\right)}}\frac{\alpha^{\left(B\right)}f_{I}^{\left(mut\right)}}{\alpha^{\left(mut\right)}}\right)$ to make the equilibrium point biologically meaningful.

### 3.2. Analysis of the Disease-Free Equilibrium Point

The system’s behavior close to the equilibrium points was determined through linear stability analysis and bifurcations. It was carried out by calculating the Jacobian matrix of the model in equilibrium points. The Jacobian matrix for (1)–(7) is given by Equation (A1) in the Appendix A. The disease-free equilibrium of the model is the steady-state solution of the model in the absence of the disease. The eigenvalues of the Jacobian matrix (A1) at this point were calculated as follows:

(46) $$\begin{array}{cc} \lambda_{1} & =0 \end{array}$$

(47) $$\begin{array}{cc} \lambda_{2} & =-\left(\alpha^{\left(B\right)}-\gamma \right) \end{array}$$

(48) $$\begin{array}{cc} \lambda_{3} & =-\left(\alpha^{\left(B\right)}-\beta \right) \end{array}$$

(49) $$\begin{array}{cc} \lambda_{4} & =-\gamma \left({f_{I}}^{\left(wt\right)}-\frac{\alpha^{\left(wt\right)}}{\alpha^{\left(B\right)}}\right) \end{array}$$

(50) $$\begin{array}{cc} \lambda_{5} & =-\gamma \left({f_{I}}^{\left(mut\right)}-\frac{\alpha^{\left(mut\right)}}{\alpha^{\left(B\right)}}\right) \end{array}$$

(51) $$\begin{array}{cc} \lambda_{6} & =-\beta \left(1-\frac{\alpha^{\left(wt\right)}}{\alpha^{\left(B\right)}}\right) \end{array}$$

(52) $$\begin{array}{cc} \lambda_{7} & =-\beta \left(1-\frac{\alpha^{\left(mut\right)}}{\alpha^{\left(B\right)}}\right) \end{array}$$

in which it is assumed that $\alpha^{\left(wt\right)}<\alpha^{\left(B\right)}$ and $\alpha^{\left(mut\right)}<\alpha^{\left(B\right)}$. Based on the general compartmental model describing an infectious disease transmission within a heterogeneous population, the host population is grouped into two general classes: the infected and uninfected compartments. Therefore, the system (1)–(7) is divided into two infected and uninfected compartments.

In classical epidemic models, it is common to observe that a disease-free equilibrium loses its stability for $R_{0}=1$, and a transcritical bifurcation occurs. A transcritical bifurcation is when a fixed point exists for all parameter values and is never destroyed. However, as the parameter values vary, this fixed point transitions from a stability region to an instability region. Biologically speaking, as $R_{0}<1$, the disease-free equilibrium would stay stable. Thus, only the commensal bacteria persist, and wt and mut *Yersinia* strains cannot pass the invasion boundary. Therefore, as $R_{0}$ increases, wt and mut *Yersinia* strains, fed by commensal bacteria, can appear. We mathematically analyze this aspect within the structure of the model to assess which parts of the structure might be responsible for the occurrence of the transcritical bifurcation.

Let us consider system (1)–(7) with the above-calculated eigenvalues. Using the eigenvectors (A9) shown in the Appendix A as a basis for a new coordinate system, we set the transformation matrix whose columns are the eigenvectors; $T={\left[v_{1},v_{2},v_{3},v_{4},v_{5},v_{6},v_{7}\right]}^{t}$. We thus have

$$\begin{array}{ccccc} \left(\begin{array}{c} B_{M}\\ Y_{M}^{\left(wt\right)}\\ Y_{M}^{\left(mut\right)}\\ B_{L}\\ Y_{L}^{\left(wt\right)}\\ Y_{L}^{\left(mut\right)} \end{array}\right)=\left(\begin{array}{c} T \end{array}\right)\left(\begin{array}{c} u_{1}\\ u_{2}\\ u_{3}\\ u_{4}\\ u_{5}\\ u_{6}\\ u_{7} \end{array}\right)\; & \; & \mathrm{and}\; & \; & \left(\begin{array}{c} u_{1}\\ u_{2}\\ u_{3}\\ u_{4}\\ u_{5}\\ u_{6}\\ u_{7} \end{array}\right)=\left(\begin{array}{c} T^{-1} \end{array}\right)\left(\begin{array}{c} B_{M}\\ Y_{M}^{\left(wt\right)}\\ Y_{M}^{\left(mut\right)}\\ B_{L}\\ Y_{L}^{\left(wt\right)}\\ Y_{L}^{\left(mut\right)} \end{array}\right). \end{array}$$

Under this transformation, the system (1)–(7) changes to

(53) $$\begin{array}{c} \left(\begin{array}{c} {\dot{u}}_{1}\\ {\dot{u}}_{2}\\ {\dot{u}}_{3}\\ {\dot{u}}_{4}\\ {\dot{u}}_{5}\\ {\dot{u}}_{6}\\ {\dot{u}}_{7} \end{array}\right)=\left(A\right)\left(\begin{array}{c} u_{1}\\ u_{2}\\ u_{3}\\ u_{4}\\ u_{5}\\ u_{6}\\ u_{7} \end{array}\right)+\left(\begin{array}{c} f_{1}\left(u_{1},\ldots ,u_{7}\right)\\ f_{2}\left(u_{1},\ldots ,u_{7}\right)\\ f_{3}\left(u_{1},\ldots ,u_{7}\right)\\ f_{4}\left(u_{1},\ldots ,u_{7}\right)\\ f_{5}\left(u_{1},\ldots ,u_{7}\right)\\ f_{6}\left(u_{1},\ldots ,u_{7}\right)\\ f_{7}\left(u_{1},\ldots ,u_{7}\right) \end{array}\right) \end{array}$$

where

$$\begin{array}{c} A=\left(\begin{array}{ccccccc} 0 & 0 & 0 & 0 & 0 & 0 & 0\\ 0 & -\frac{\gamma \left(\alpha^{\left(B\right)}{f_{I}}^{\left(wt\right)}-\alpha^{\left(wt\right)}\right)}{\alpha^{\left(B\right)}} & 0 & 0 & 0 & 0 & 0\\ 0 & 0 & -\frac{\gamma \left(\alpha^{\left(B\right)}{f_{I}}^{\left(mut\right)}-\alpha^{\left(mut\right)}\right)}{\alpha^{\left(B\right)}} & 0 & 0 & 0 & 0\\ 0 & 0 & 0 & -\frac{\beta \left(\alpha^{\left(B\right)}-\alpha^{\left(wt\right)}\right)}{\alpha^{\left(B\right)}} & 0 & 0 & 0\\ 0 & 0 & 0 & 0 & -\frac{\beta \left(\alpha^{\left(B\right)}-\alpha^{\left(mut\right)}\right)}{\alpha^{\left(B\right)}} & 0 & 0\\ 0 & 0 & 0 & 0 & 0 & -\alpha^{\left(B\right)}+\beta & 0\\ 0 & 0 & 0 & 0 & 0 & 0 & -\alpha^{\left(B\right)}+\gamma \end{array}\right) \end{array}$$

so that the linear part is now in a standard (diagonal) form. In the $\left(u_{1},u_{2},u_{3},u_{4},u_{5},u_{6},u_{7}\right)$ coordinates, the center manifold is a curve tangent to the $u_{1}-\mathrm{axis}$. The projection of the system onto the $u_{1}-\mathrm{axis}$ is obtained by setting $u_{2}=u_{3}=u_{4}=u_{5}=u_{6}=u_{7}=0$ in the equation for ${\dot{u}}_{1}$. It yields ${\dot{u}}_{1}=0$. Since $E^{c}$ is one dimension, we can approximate $h_{i}\left(u_{1}\right)$ by Taylor expansion such that $u_{i}=h_{i}\left(u_{1}\right)$, i∈{2,…,7}, satisfying the following equations:

(54) $$\begin{array}{c} \begin{array}{ccc} {\dot{u}}_{2} & = & Dh_{2}\left(u_{1}\right){\dot{u}}_{1},\\ {\dot{u}}_{3} & = & Dh_{3}\left(u_{1}\right){\dot{u}}_{1},\\ {\dot{u}}_{4} & = & Dh_{4}\left(u_{1}\right){\dot{u}}_{1},\\ {\dot{u}}_{5} & = & Dh_{5}\left(u_{1}\right){\dot{u}}_{1},\\ {\dot{u}}_{6} & = & Dh_{6}\left(u_{1}\right){\dot{u}}_{1},\\ {\dot{u}}_{7} & = & Dh_{7}\left(u_{1}\right){\dot{u}}_{1},\\ h_{i}\left(0\right) & = & Dh_{i}\left(0\right)=0 \end{array} \end{array}$$

Thus, the reduced system is

(55) $${\dot{u}}_{1}=-\frac{\alpha^{\left(B\right)}}{C_{M}}\;u_{1}^{2}+O\left(u_{1}^{3}\right).$$

The advantages of a center manifold are clear from this calculation. We may study a one-dimensional system instead of a seven-dimensional system. That is, the method of center manifolds enables one to reduce the dimensions of the system by studying the flow restricted to the center manifold, in which the transients associated with the nonzero eigenvalues have decayed. As long as $\alpha^{\left(wt\right)}<\alpha^{\left(B\right)}$ and $\alpha^{\left(mut\right)}<\alpha^{\left(B\right)}$, the disease-free equilibrium point will persist. When the infection rate of wt and mut strains increases, the system becomes unstable at the disease-free equilibrium point, and the trajectory of the system approaches asymptotically to the bacterial population of wt or mut and, finally, to the co-existence of the strains.

### 3.3. Computing and Analysis of the System through the Basic Reproduction Number

For scenarios where multiple strains (subtypes) of an infectious disease exist, an $R_{0}$ number is calculated for each strain. Hence, in our model, we must define two different $R_{0}$, one for the wt and the other for the mut strain, rather than an $R_{0}$ for the whole model. This is a significantly more difficult task. Herein, we introduce the following variations to the basic reproduction number, which are calculated by the average number of secondary infections:

1. $R_{0}^{wt}$: the basic reproductive numbers for wt strain = $\frac{\alpha^{\left(wt\right)}}{\alpha^{\left(B\right)}f_{I}^{\left(wt\right)}}$
2. $R_{0}^{mut}$: the basic reproductive numbers for mut strain = $\frac{\alpha^{\left(mut\right)}}{\alpha^{\left(B\right)}f_{I}^{\left(mut\right)}}$.

To understand the role of the basic reproduction number, we also define a single reproduction number for commensal bacteria $R_{0}^{com}$, computed as an expected number of secondary cases of commensal bacteria produced by a single commensal bacteria:

(56) $$R_{0}^{com}=\max\left\{\frac{\alpha^{\left(B\right)}}{\beta },\frac{\alpha^{\left(B\right)}}{\gamma }\right\}.$$

Due to biologically meaningful disease-free state $P^{0}$ and holding the conditions $\beta <\alpha^{\left(B\right)}$ and $\gamma <\alpha^{\left(B\right)}$, the disease-free state would be meaningful if $R_{0}^{com}>1$. Therefore, the commensal bacteria appear, $R_{0}^{com}$ slightly reaches above one, and a stable disease-free state happens. Due to the presence of commensal bacteria and instability of the trivial solution, the solution corresponds to the extinction of all populations that never appear because if $R_{0}^{com}<1$, all components of the disease-free equilibrium will lie out of the biologically meaningful region.

For $R_{0}^{wt}<1$ and $R_{0}^{mut}<1$, thus, $\lambda_{1}=0$ is a simple zero eigenvalue, and the other eigenvalues are real and negative. This implies that transcritical bifurcation occurs in the disease-free equilibrium for $R_{0}^{wt}<1$ and $R_{0}^{mut}<1$, and the uninfected state is stable (not asymptotically stable) as none of the strains persist. As soon as the two equilibria collide non-destructively, exchanging their stability and resulting in the Jacobian matrix having a single eigenvalue that is equal to zero, then either the mut strain or the wt appears and persists for $R_{0}^{mut}$ or $R_{0}^{wt}$, respectively.

**Figure 2:**
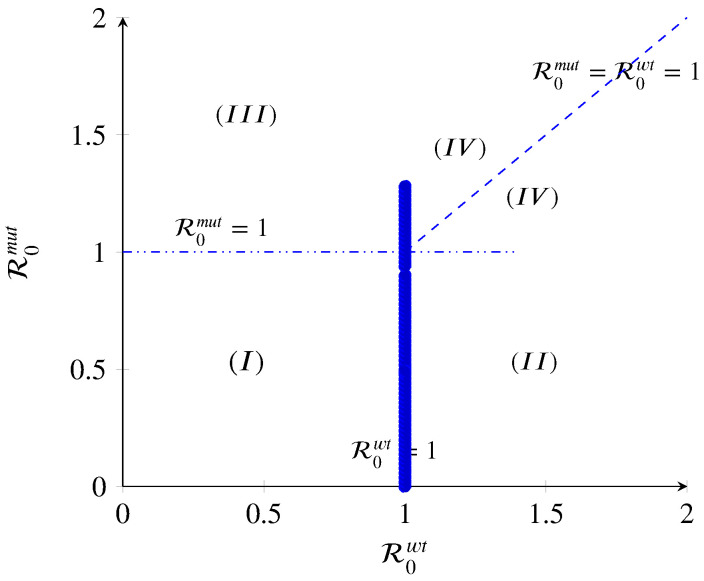
The diagram displays three regions with different qualitative behaviors in terms of the basic reproduction numbers. Region I: infection-free state; Region II: mut-free state; Region III: wt-free state; Region IV: co-existence of all strains.

As shown in Figure 2, when the disease-free equilibrium loses its stability, different scenarios can occur. Herein, we discuss the different cases.

(I) If $R_{0}^{wt}=1$ and $R_{0}^{mut}=1$, then $\alpha^{\left(wt\right)}=\alpha^{\left(B\right)}f_{I}^{\left(wt\right)}$ and $\alpha^{\left(mut\right)}=\alpha^{\left(B\right)}f_{I}^{\left(mut\right)}$. Thus, the intersection of the transcritical curves $R_{0}^{wt}$ and $R_{0}^{mut}$ results in a triple transcritical bifurcation. As shown in (A9), the Jacobian has a triple zero eigenvalue at this point ($\lambda_{2}=0,\lambda_{3}=0$). Kuznetsov [34] has proved that such a point would be an indicator of the onset of a non-degenerate or degenerate Bogdanov–Takens bifurcation [34,35]. The disease-free equilibrium $P^{0}$ loses its stability, and the wt-free and mut-free include one simple zero eigenvalue ($\lambda_{3}=0$), meaning that the dynamics of the model change as the target parameter is within the threshold value.
(II) If $R_{0}^{wt}>1$, the wt strain equilibrium in region II will persist when $R_{0}^{mut}<1$. The wt strain will spread and possibly persist within the host population. In general, for a strain to persist, its basic reproduction number has to be strictly greater than one. Therefore, in this region, the disease-free, mut strain, and co-existence state exchange stability: $P^{0}$ becomes unstable, $P^{2}$ becomes locally asymptotically stable, and $P^{1}$ and $P^{3}$ remain unstable. This means that the immune system could kill one of the strains more efficiently.
(III) If $R_{0}^{mut}>1$, the mut strain equilibrium in region III will persist when $R_{0}^{wt}<1$. The mut strain will spread and possibly persist within the host population since its basic reproduction number is greater than one. Therefore, in this region, the disease-free, wt strain, and co-existence state exchange stability: $P^{0}$ becomes unstable, $P^{1}$ becomes locally asymptotically stable, and $P^{2}$ and $P^{3}$ remain unstable. This means that the immune system could defeat the wt strains. However, the risk of this situation to happen is low because the mut strains are influenced more efficiently than wt strains by immune action.
(IV) If $R_{0}^{wt}>1$ and $R_{0}^{mut}>1$, the co-existence population spreads, and both strains persist. The overall $R_{0}$ can be defined as $R_{0}^{c}=d\;R_{0}^{mut}+\left(1-d\right)\;R_{0}^{wt}$. A mut with d=1 is thoroughly dominant, while one with d=0 is completely recessive; scenarios of incomplete dominance (0<d<1), under-dominance (d<0), and over-dominance (d>1) are possible as well. For instance, a mut could achieve a higher $R_{0}$ than the wt via a higher growth rate that increases transmission. In a co-infection, the faster-growing mut strain would outcompete the wt and reach its maximum capacity. This situation would change the co-infection to the conditions where a single infection happens. Thus, the overall $R_{0}$ of the co-infection would be similar to that of the mut by itself, making the mut a dominant one. Furthermore, the effort for having a co-existence equilibrium and analysis of the co-infection model will fail. By contrast, let us assume a mut strain achieves a higher $R_{0}$. Nevertheless, the virulence of the wt strain neutralizes the higher $R_{0}$ value of the mut. This would make the mut a recessive one. In summary, virtually any two-strain co-infection model can be mapped to a set of values for *d*, allowing scenarios of particular interest to be explored in a context broader than the one possible with typical models.

The mathematical model (1)–(7) calculates the number of pathogen-specific characteristics in different layouts, e.g., when colonization resistance mediated by the endogenous microbiome is lacking or when the immune response is partially impaired. In this paper, we use the experimental data obtained in the lab upon infection of a host either lacking a microbiome (mimicking antibiotic treatment of patients) or a fully functional immune system [10].

To challenge the numerical simulation with experimental data, we tested whether the numerical simulation from the mathematical analysis could fit the experimental data.

Correspondingly, we simulated the process of *Yersinia enterocolitica* co-infection in specific-pathogen-free (SPF) (i.e., wt), germ-free (GF), and MyD88-deficient (MyD88^−/−^) mut mice. Using the model values in Table 3, which was estimated through experimental data [10], we first examined the influence of the infection rates $\alpha^{\left(B\right)}$, $\alpha^{\left(wt\right)}$, and $\alpha^{\left(mut\right)}$ on the dynamics beginning by constructing one-dimensional bifurcation diagrams using $\alpha^{\left(B\right)}$ as the bifurcation parameter and fixing $\alpha^{\left(wt\right)}$ and $\alpha^{\left(mut\right)}$. The model values in Table 3 are the output of estimation parameters in Table 2 of the (1)–(7) by experimental data [10]. In theory, some parameter values were defined based on the biological aspect, and the rest were estimated by the optimization problem with the maximum likelihood estimation [10].

**Figure 3:**
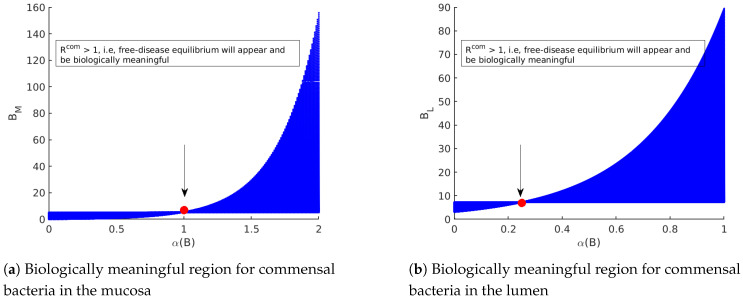
The diagrams display the role of $R_{0}^{com}$ in appearance/non-appearance of the trivial solution and disease-free equilibrium. (**a**) By fixing the parameter values Table 3 in model (14), the commensal bacteria in the mucosa appear when $R_{0}^{com}>1$. Therefore, as long as $R_{0}^{com}<1$, only the trivial solution for the model exists. Since the trivial solution is always unstable, the extinction of all populations is never achieved. (**b**) By fixing the parameter values Table 3 in model (14), the commensal bacteria in the lumen appear when $R_{0}^{com}>1$. Therefore, as long as $R_{0}^{com}<1$, only the trivial solution for the model exists. Since the trivial solution is always unstable, the extinction of all populations is never achieved.

As shown in Figure 3, $R_{0}^{com}$ should be larger than one to achieve the disease-free equilibrium. As long as $R_{0}^{wt}$ and $R_{0}^{mut}$ are smaller than one, the disease-free equilibrium is stable.

Following the analysis of the model with respect to $\alpha^{\left(B\right)}$, two cases are considered. The first case is when $\alpha^{\left(wt\right)}$ and $\alpha^{\left(mut\right)}$ are equal. In this case, we do not have co-existence of wt and mut (the denominators of $Y_{L}^{3(wt)}$ and $Y_{L}^{3(mut)}$ will be equal to zero). The second one is when $\alpha^{\left(wt\right)}$ and $\alpha^{\left(mut\right)}$ have different values as the co-existence equilibrium is achieved and is biologically meaningful.

Thus, we start with values of the wt and mut-strain infections rate given by $\alpha^{\left(wt\right)}=4.44E-01$ and $\alpha^{\left(wt\right)}=4.44E-01$ in Table 3. Corresponding to those infections rates, the basic reproduction numbers ${R_{0}}^{\left(wt\right)}$ and ${R_{0}}^{\left(mut\right)}$ in terms of $\alpha^{\left(B\right)}$ are computed.

**Figure 4:**
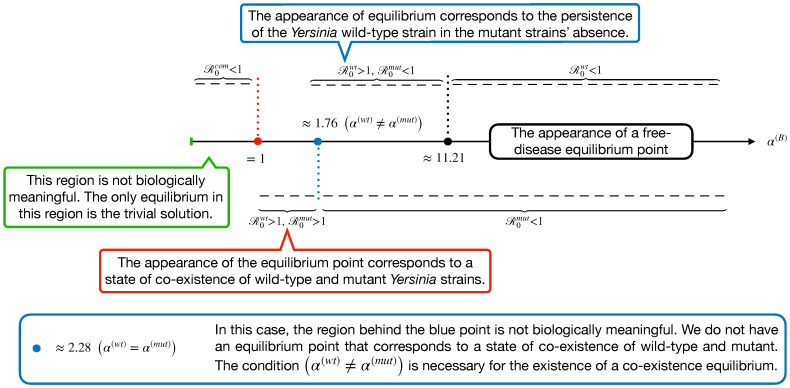
The diagram displays $\alpha^{\left(B\right)}$ regions with the appearance of a different equilibrium in terms of the basic reproduction numbers. This display is plotted by fixing the parameter values from Table 3 in Model (14).

Through the definitions of $R_{0}^{com}$, $R_{0}^{wt}$, and $R_{0}^{mut}$, we display an *x*-scale as $\alpha^{\left(B\right)}$ in Figure 4. This shows the appearance of a different equilibrium of Model (14) in terms of the basic reproduction numbers.

**Figure 5:**
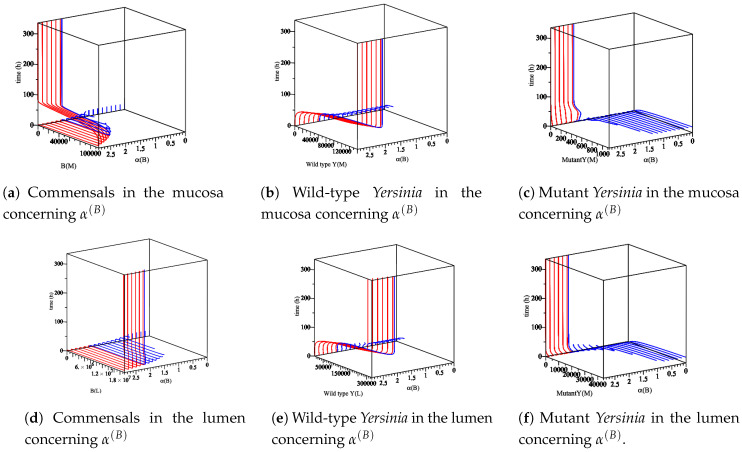
The sensitivity of parameter $\alpha^{\left(B\right)}$ with respect to commensal bacteria, wild-type, and mutant *Yersinia* in the mucosa and lumen for 336 h. $0<\alpha^{\left(B\right)}<1$, no biologically meaningful region (out of our interest). $\alpha^{\left(B\right)}>1$, all populations appear. When $1<\alpha^{\left(B\right)}<2.28$, the region is a region for the appearance of the co-existence equilibrium, but the hypothesis of the co-existence equilibrium is not satisfied. Therefore, wild-type strain does not grow, and the mutant strain is going down slowly. When $\alpha^{\left(B\right)}>2.28$, this is a region of the appearance of a wt equilibrium $R_{0}^{wt}>1$. Thus, (**a**,**e**) are increasing fast and stay at the maximum level as (**a**,**d**) are going back to zero. Additionally, (**c**,**d**) do not grow anymore $R_{0}^{mut}<1$.

As shown in Figure 4, we expect no co-existence equilibrium when $\alpha^{\left(wt\right)}=\alpha^{\left(mut\right)}$, and only wt equilibrium exists (mut-free equilibrium) when $1.76<\alpha^{\left(B\right)}<11.20$. However, the disease-free equilibrium can happen when $\alpha^{\left(B\right)}$ is large enough to surpass $\alpha^{\left(wt\right)}$ and $\alpha^{\left(mut\right)}$. These findings are displayed in Figure 5.

**Figure 6:**
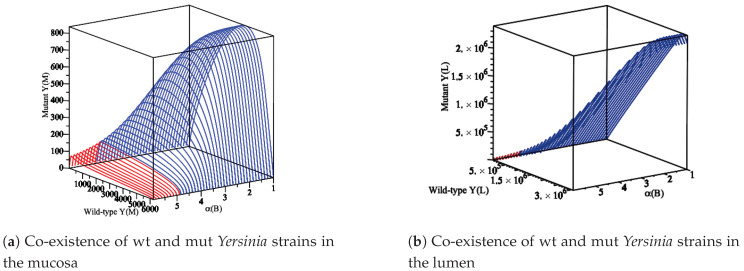
The diagram displays the appearance of the co-existence of wt and mut *Yersinia* strains as $\alpha^{\left(B\right)}$ is changing in a region where $R_{0}^{wt}>1$ and $R_{0}^{mut}>1$. (**a**) An immune reaction influences the wt and mut Yersinia strains in the mucosa. Additionally, another part is spilled over and moves to the lumen compartment. Therefore, they simultaneously increase or decrease to reach their maximum values. (**b**) The wt and mut Yersinia strains in the lumen simultaneously increase to reach the carrying capacity of the lumen compartment. In both (**a**,**b**), when $\alpha^{\left(B\right)}$ reaches 4.90, the co-existence of wt and mut *Yersinia* strains disappears.

Secondly, to show the appearance of the co-existence, we consider the parameter values of in Ye wt/T3S0 from Table 3 with the assumption of $1.86=\alpha^{wt}\neq \alpha^{mut}=1.83$. Thus, we compute $R_{0}^{wt}$ and $R_{0}^{mut}$ with respect to parameter $\alpha^{\left(B\right)}$. This results in the appearance co-existence when $1<\alpha^{\left(B\right)}<4.90$ as Figure 6 shows.

**Figure 7:**
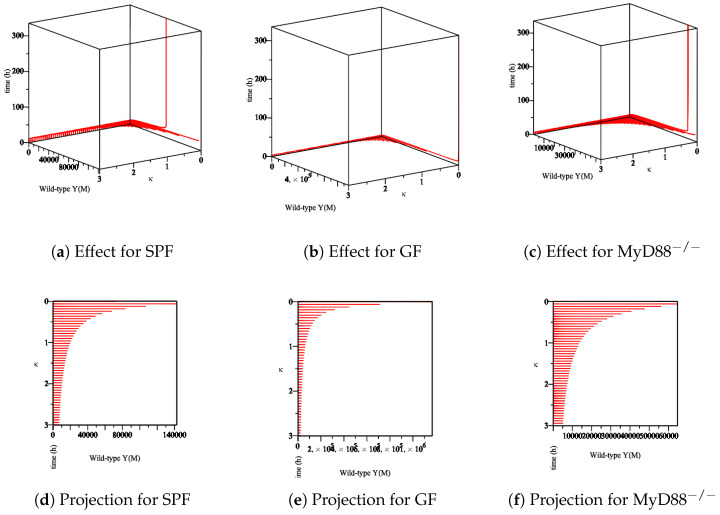
The sensitivity of parameter *κ* in the elimination of wt *Yersinia* strain in the mucosa. (**a**–**c**) The effect of the parameter *κ* on the different types of mice. (**d**–**f**) The projection of *κ* for a different types of mice.

**Figure 8:**
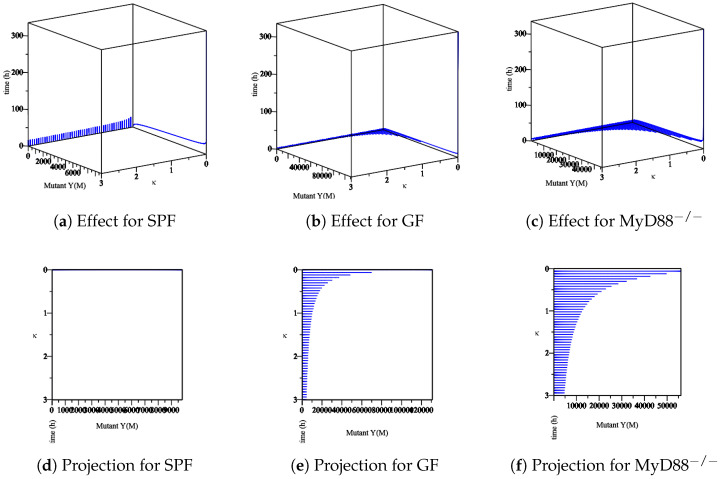
The sensitivity of parameter *κ* in the elimination of mut *Yersinia* strain in the mucosa. (**a**–**c**) The effect of the parameter *κ* on the different types of mice. (**d**–**f**) The projection of *κ* for different types of mice.

**Figure 9:**
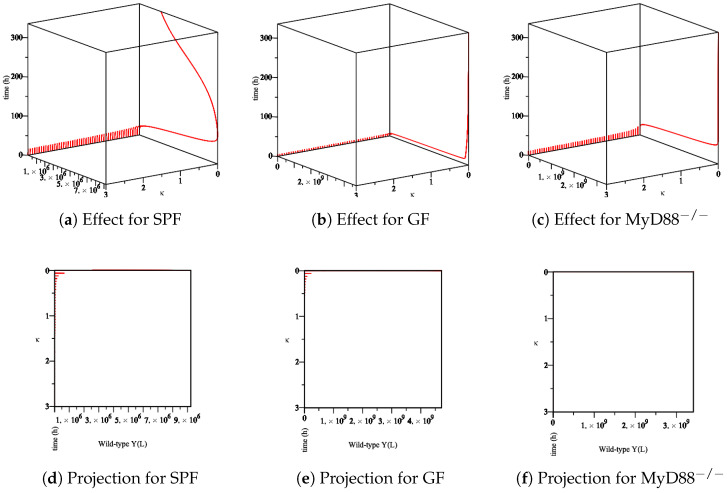
The sensitivity of parameter *κ* in the elimination of wt *Yersinia* strain in the lumen. (**a**–**c**) The effect of the parameter *κ* on the different types of mice. (**d**–**f**) The projection of *κ* for different types of mice.

**Figure 10:**
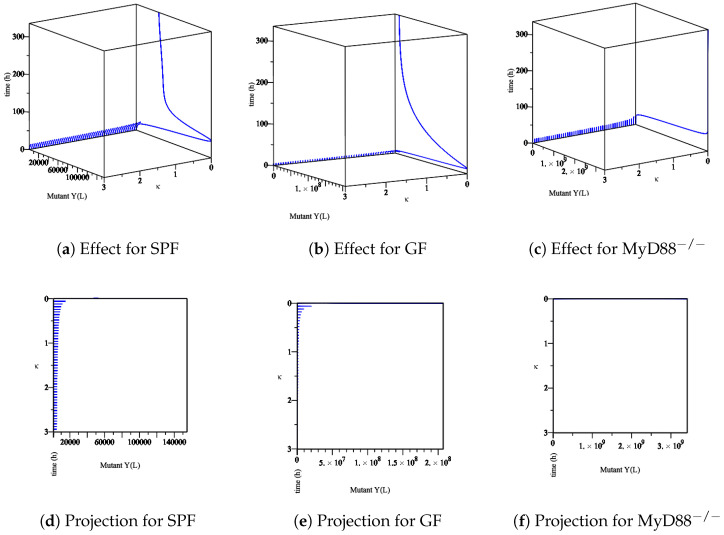
The sensitivity of the parameter *κ* in the elimination of the mut *Yersinia* strain in the lumen. (**a**–**c**) The effect of the parameter *κ* on the different types of mice. (**d**–**f**) The projection of *κ* for different types of mice.

Furthermore, we analyze the immune system’s influence. In our model, at least one Ye within the mucosal compartment activates the immune system. This activation increases proportionally to the number of Ye. The immune system is assumed to influence the bacterial populations primarily at the mucosal site compared to bacteria within the lumen. Herein, we simulate the process of *Yersinia enterocolitica* with an immune response that was experienced in SPF, GF, and MyD88^−/−^ mice. As the responses of the immune system in these mice are different, we conclude different behaviors. Let us denote the numerical simulation:

- The effect of the maximum rate of immune growth κ on wt *Yersinia* strain in the mucosa, Figure 7;
- The effect of the maximum rate of immune growth κ on mut *Yersinia* strain in the mucosa, Figure 8;
- The effect of the maximum rate of immune growth κ on wt *Yersinia* strain in the lumen, Figure 9;
- The effect of the maximum rate of immune growth κ on mut *Yersinia* strain in the lumen, Figure 10,

The experimental data obtained in the lab showed that the Ye mut strain was eliminated more efficiently than Ye wt in the mucosal compartment. However, since GF mice lack a microbiome, both the Ye wt and the mut strains can colonize the GIT at high numbers. In the SPF-colonized MyD88 mice, the immune response is weaker [10]. Therefore, as shown in Figure 7, Figure 8, Figure 9 and Figure 10, the following results conclude:

- Figure 7d and Figure 8d show a slow reduction in the wt strain in comparison to mut strain as κ changes.
- Figure 7b, Figure 8b, Figure 9b and Figure 10b in comparison with similar situations in SPF and MyD88^−/−^ show elimination of wt and mut strains is less efficient.
- Figure 7c, Figure 8c, Figure 9c and Figure 10c show that wt and the mut strains increased faster. Besides, the influence of κ on wt and the mut strains cannot project properly (Figure 9f and Figure 10f) at the same speed of producing strains.

**Figure 11:**
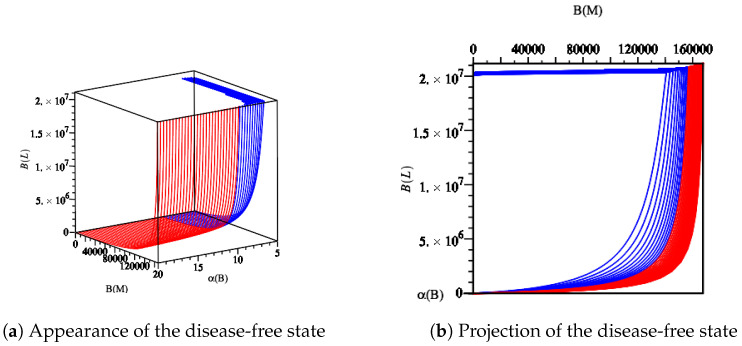
Diagram displaying the disease-free state by fixing the parameter values Ye SPF wt/A0 from Table 3 in Model (14). As long as $\alpha^{\left(B\right)}$ is large enough, the disease-free state persists. However, by reducing $\alpha^{\left(B\right)}$, the basic reproduction number $R_{0}^{wt}$ reaches its threshold value ($R_{0}^{wt}=1$). This causes changes in the dynamic behavior of the model (14), as shown in Figure 4.

Our results allow us to state that if mice possess a healthy microbiota and immune system, as long as the growth rate of commensal bacteria is larger than the growth rate of wt and mut *Yersinia*, then the infection will not spread, as Ye strains cannot enter the lumen compartment. Therefore, the disease-free equilibrium, $P^{0}$ exists and is stable when $R_{0}^{wt}$ and $R_{0}^{mut}$ are less than one while $\alpha^{\left(B\right)}$ is large enough. To challenge this situation, we tested the data values Ye SPF wt/A0 from Table 3 with the assumed boundary $\alpha^{\left(B\right)}>11.20$ to face a disease-free state, Figure 4. Our computer simulations ran for 366 h and are shown in Figure 11. As predicted for the disease-free state, the commensal fractions $B_{M}$ and $B_{L}$ approached their carrying capacities $C_{M}$ and $C_{L}$, respectively.

Therefore, we note that the disease-free equilibrium always exists and is (asymptotically) stable whenever the reproductive numbers for both strains of the disease are below unity. Simulations support these findings. The two single-strain equilibria, where one strain persists while the other strain dies out, are symmetrical and exist when at least one of the strains has a reproductive number above unity. However, in our model, (14) with parameter values from Table 3, we could not observe a state that corresponds to Ye mut strain equilibrium. As shown in Figure 4, there is no region where $R_{0}^{mut}>1$ and $R_{0}^{wt}<1$. Whenever $R_{0}^{mut}>1$, $R_{0}^{wt}$ is already above one; this facilitates the situation for a co-existence equilibrium. This is where both strains persist, existing when both reproductive numbers are above a certain threshold. However, we could not analytically solve the stability criteria for the equilibrium due to complexity, but simulations show that these stability criteria exist.

## 4. Discussion and Conclusions

In this paper, we proved that our computational model of the *Yersinia enterocolitica* [10] population in co-infections of mice with Ye wt and mut strains always showed stable results in terms of $R_{0}$, such that if we can keep or reduce $R_{0}s$ to less than one, then wt and mut strains cannot spread, and the infection resolves. The medical control strategies for this infectious disease, like other infectious diseases, are frequently predicted through the basic reproduction number. To know what the difference is, we should be aware that the decomposition in dynamics and the designation of compartments as infected or uninfected are unique, and the basic reproduction number is achieved in the same way for all different compartmental models. This also raises the question of how the infection becomes persistent or is resolved based on the calculation of $R_{0}$ as a significant measure in control policy.

The answer to this question depends on the magnitude of the basic reproductive number, $R_{0}$, since calculating the stability of an equilibrium is very complicated. Therefore, we computed $R_{0}$ for our computational model based on different strains. This resulted in three $R_{0}s$: one $R_{0}^{com}$, $R_{0}^{wt}$, and $R_{0}^{mut}$.

As it is obvious in any compartmental model, the disease-free equilibrium is locally (asymptotically) stable when $0<R_{0}<1$ and unstable if $R_{0}>1$. In other words, when $0<R_{0}<1$, every infectious strain will cause less than one secondary infection; hence, the strain will eliminate. When $R_{0}>1$, every infectious strain will cause more than one secondary infection; hence, *Yersinia* infection strains will persist. However, all public health control measures can usually be based on methods that, if practical, would lower $R_{0}$ to below unity. On the other hand, the co-infection equilibrium is locally stable when $R_{0}>1$ and unstable when $0<R_{0}<1$.

This trivial result is essential, but we had to adjust our multi-strain model with different $R_{0}s$. To more smoothly analyze $R_{0}s$, we looked for a common parameter in all $R_{0}s$. This resulted in the parameter $\alpha^{\left(B\right)}$. We thus analyzed at which level of sensibility the system’s parameter $\alpha^{\left(B\right)}$ changes the model’s invasion behavior. This came to an end when a disease-free state occurs, when $\alpha^{\left(B\right)}$ satisfies the conditions $\beta <\alpha^{\left(B\right)}$ and $\gamma <\alpha^{\left(B\right)}$. In addition, if $\alpha^{\left(B\right)}$ is large enough, then the disease-free state can be stable, and the infection with other strains does not appear or spread. A mut strain equilibrium does not occur for parameter values estimated through experimental data [10] since there is no region with only $R_{0}^{mut}>1$. Although there is a region with two threshold parameters in the model, $R_{0}^{wt}$ and $R_{0}^{mut}$ stay above one for a co-infection setup. This region includes a small range for the parameter $\alpha^{\left(B\right)}$. In addition, it is important that the maximum growth rates associated with the wt and mut strains should be unequal. Otherwise, the co-existence scenario cannot take place.

Furthermore, we looked for conditions in the immune system to see different scenarios of a weaker and more robust immune system in fighting against infection. This resulted in the analysis of systems with respect to parameter κ. We found that if mice possess a healthy microbiota and immune system, as long as the growth rate of commensal bacteria is larger than the growth rate of wt and mut *Yersinia*, then the infection will not spread, as Ye strains cannot enter the lumen compartment.

Our results indicate that the within-host dynamics of immunity can, in principle, have significant consequences on population-level dynamics. However, immunity alone never creates a backward bifurcation of the disease-free steady state under biologically realistic hypotheses, and this would require some other complementary conditions.

Altogether, *Yersinia* is a great model system and can be used to predict the spread of infections for more clinically relevant bugs, i.e., enteropathogenic or enterohemorrhagic *Escherichia coli*. To control the spread of infection, efforts must be made to ensure that the co-infection equilibrium is unstable, i.e., $0<R_{0}<1$, and in our model $R_{0}^{wt}<1$ and $R_{0}^{mut}<1$.

Furthermore, an additional factor to take into account is whether antibiotics are used from the beginning of the infection, in which case the spread of *Yersinia* may or may not be extinguished. We also believe that further research into the elaborate makeup of the strains and how the immune system differentiates between strains could be incorporated to more accurately represent control policy.

## Figures and Tables

**Table 1:** The variable symbols and their meaning. The lumen is abbreviated with *L*, the mucosa as *M*. We also indicate each variable with mut or wt to denote to which population they refer. An upper-case *I* refers to the immune system. SBML [26,27] defines the units *item* and *dimensionless* to indicate that a quantity occurs in a piece number or that its unit originates from the cancellation of other units.

Variable Symbol	Meaning	Units
$B_{M}$	Commensal bacteria in the mucosa	item
$Y_{M}^{\left(wt\right)}$	wt *Yersinia* in the mucosa	item
$Y_{M}^{\left(mut\right)}$	mut *Yersinia* in the mucosa	item
$B_{L}$	Commensal bacteria in the lumen	item
$Y_{L}^{\left(wt\right)}$	wt *Yersinia* in the lumen	item
$Y_{L}^{\left(mut\right)}$	mut *Yersinia* in the lumen	item
*I*	Strength of immune reaction	dimensionless

**Table 2:** Definition of the parameters.

Parameter	Definition	Unit
$\alpha^{\left(B\right)}$	Maximal growth rate of intestinal bacteria	1/h
$\alpha^{\left(wt\right)}$	Maximal growth rate of wt *Yersinia*	1/h
$\alpha^{\left(mut\right)}$	Maximal growth rate of mut *Yersinia*	1/h
$f_{I}^{\left(wt\right)}$	Immunity adjustment factor for wt *Yersinia*	dimensionless
$f_{I}^{\left(mut\right)}$	Immunity adjustment factor for mut *Yersinia*	dimensionless
$C_{M}$	Carrying capacity of the mucosa	item
$C_{L}$	Carrying capacity of the lumen	item
$C_{I}$	Carrying capacity of the immune system	item
γ	Maximal immunity action	1/h
κ	Maximal rate of immune growth	1/h
β	Rate at which intestines are discharged	1/h

**Table 3:** Parameter values.

Parameter	Values in Ye SPF wt/A0	Values in Ye SPF wt/T3S0	Values in Ye GF wt/A0	Values in Ye MyD88^−/−^ wt/A0
$\alpha^{\left(B\right)}$	4.89 × 10^−1^	2.00	1.99	5.40 × 10^−1^
$\alpha^{\left(wt\right)}$	4.44 × 10^−1^	1.86	1.60	5.78 × 10^−1^
$\alpha^{\left(mut\right)}$	4.44 × 10^−1^	1.86	1.60	5.78 × 10^−1^
$f_{I}^{\left(wt\right)}$	3.96 × 10^−1^	9.48 × 10^−3^	1.10 × 10^−1^	6.23 × 10^−2^
$f_{I}^{\left(mut\right)}$	1.95 × 10^−1^	3.73 × 10^−1^	1.19 × 10^−1^	1.28 × 10^−1^
$C_{M}$	1.76 × 10^5^	6.27 × 10^3^	1.3 × 10^6^	1.28 × 10^5^
$C_{L}$	2.14 × 10^7^	6.13 × 10^6^	4.99 × 10^9^	9.98 × 10^9^
$C_{I}$	1.00	1.00	1.00	1.00
γ	1.00	1.00	9.97 × 10^−1^	1.00 × 10^−1^
κ	7.83 × 10^−1^	4.28 × 10^−1^	6.50 × 10^−1^	4.37 × 10^−1^
β	2.50 × 10^−1^	2.50 × 10^−1^	8.33 × 10^−2^	1.82 × 10^−1^

## Abbreviations

AMP	antimicrobial peptide
BMBF	Federal Ministry of Education and Research
BMBF-DZG	*Deutsche Zentren der Gesundheitsforschung*
CFU	colony-forming unit
DFE	disease-free equilibrium
DFG	*Deutsche Forschungsgemeinschaft*
DZIF	German Center for Infection Research
GF	germ-free
GIT	gastrointestinal tract
mut	mutant
MyD88^−/−^	MyD88-deficient mice
ODE	Ordinary Differential Equation
PD	Process Description
RKI	Robert Koch Institute
SBGN	Systems Biology Graphical Notation
SI	small intestine
SPF	specific-pathogen-free
T3SS	Type iii@ secretion system
wt	wild-type
YadA	Yersinia adhesin A
Ye	*Yersinia entercolitica*
Yop	*Yersinia* outer protein

## Appendix A. Mathematical Calculation

### Appendix A.1. Jacobian

The Jacobian of the system (1)–(7) is calculated as follows:

(A1) $$\begin{array}{c} J=\left(\begin{array}{cc} \alpha^{\left(B\right)}\left(1-\frac{2B_{M}+Y_{M}^{\left(wt\right)}+Y_{M}^{\left(mut\right)}}{C_{M}}\right)-\gamma I & -\frac{\alpha^{\left(B\right)}B_{M}}{C_{M}}\\ -\frac{\alpha^{\left(wt\right)}Y_{M}^{\left(wt\right)}}{C_{M}} & \alpha^{\left(wt\right)}\left(1-\frac{B_{M}+2Y_{M}^{\left(wt\right)}+Y_{M}^{\left(mut\right)}}{C_{M}}\right)-\gamma If_{I}^{\left(wt\right)}\\ -\frac{\alpha^{\left(mut\right)}Y_{M}^{\left(mut\right)}}{C_{M}} & -\frac{\alpha^{\left(mut\right)}Y_{M}^{\left(mut\right)}}{C_{M}}\\ \alpha^{\left(B\right)}\frac{2B_{M}+Y_{M}^{\left(wt\right)}+Y_{M}^{\left(mut\right)}}{C_{M}} & \frac{\alpha^{\left(B\right)}B_{M}}{C_{M}}\\ \frac{\alpha^{\left(wt\right)}Y_{M}^{\left(wt\right)}}{C_{M}} & \alpha^{\left(wt\right)}\frac{B_{M}+2Y_{M}^{\left(wt\right)}+Y_{M}^{\left(mut\right)}}{C_{M}}\\ \frac{\alpha^{\left(mut\right)}Y_{M}^{\left(mut\right)}}{C_{M}} & \frac{\alpha^{\left(mut\right)}Y_{M}^{\left(mut\right)}}{C_{M}}\\ 0 & \frac{\kappa (C_{I}-I)}{C_{I}} \end{array}\right.\\ \begin{array}{cc} -\frac{\alpha^{\left(B\right)}B_{M}}{C_{M}} & 0\\ -\frac{\alpha^{\left(wt\right)}Y_{M}^{\left(wt\right)}}{C_{M}} & 0\\ \alpha^{\left(mut\right)}\left(1-\frac{B_{M}+2Y_{M}^{\left(wt\right)}+Y_{M}^{\left(mut\right)}}{C_{M}}\right)-\gamma If_{I}^{\left(mut\right)} & 0\\ \frac{\alpha^{\left(B\right)}B_{M}}{C_{M}} & \alpha^{\left(B\right)}\left(1-\frac{2B_{L}+Y_{L}^{\left(wt\right)}+Y_{L}^{\left(mut\right)}}{C_{L}}\right)-\beta \\ \frac{\alpha^{\left(wt\right)}Y_{M}^{wt}}{C_{M}} & -\frac{\alpha^{\left(wt\right)}Y_{L}^{\left(wt\right)}}{C_{L}}\\ \alpha^{\left(mut\right)}\frac{B_{M}+Y_{M}^{\left(wt\right)}+2Y_{M}^{\left(mut\right)}}{C_{M}} & -\frac{\alpha^{\left(mut\right)}Y_{L}^{\left(mut\right)}}{C_{L}}\\ \frac{\kappa (C_{I}-I)}{C_{I}} & 0 \end{array}\\ \left.\begin{array}{ccc} 0 & 0 & -\gamma B_{M}\\ 0 & 0 & -\gamma f_{I}^{\left(wt\right)}Y_{M}^{\left(wt\right)}\\ 0 & 0 & -\gamma f_{I}^{\left(mut\right)}Y_{M}^{\left(mut\right)}\\ -\frac{\alpha^{\left(B\right)}B_{L}}{C_{L}} & -\frac{\alpha^{\left(B\right)}B_{L}}{C_{L}} & 0\\ \alpha^{\left(wt\right)}\left(1-\frac{B_{L}+2Y_{L}^{\left(wt\right)}+Y_{L}^{\left(mut\right)}}{C_{L}}\right)-\beta & -\frac{\alpha^{\left(wt\right)}Y_{L}^{wt}}{C_{L}} & 0\\ -\frac{\alpha^{\left(mut\right)}Y_{L}^{\left(mut\right)}}{C_{L}} & \alpha^{\left(mut\right)}\left(1-\frac{B_{L}+Y_{L}^{\left(wt\right)}+2Y_{L}^{\left(mut\right)}}{C_{L}}\right)-\beta & 0\\ 0 & 0 & -\frac{\kappa (Y_{M}^{\left(wt\right)}+Y_{M}^{\left(mut\right)})}{C_{I}} \end{array}\right) \end{array}$$

### Appendix A.2. The Eigenvalues of Trivial Equilibrium Point

The eigenvalues for trivial equilibrium points are as follows:

(A2) $$\begin{array}{cc} \lambda_{1} & =0 \end{array}$$

(A3) $$\begin{array}{cc} \lambda_{2} & =\alpha^{\left(B\right)} \end{array}$$

(A4) $$\begin{array}{cc} \lambda_{3} & =\alpha^{\left(wt\right)} \end{array}$$

(A5) $$\begin{array}{cc} \lambda_{4} & =\alpha^{\left(mut\right)} \end{array}$$

(A6) $$\begin{array}{cc} \lambda_{5} & =\alpha^{\left(B\right)}-\beta \end{array}$$

(A7) $$\begin{array}{cc} \lambda_{6} & =\alpha^{\left(wt\right)}-\beta \end{array}$$

(A8) $$\begin{array}{cc} \lambda_{7} & =\alpha^{\left(mut\right)}-\beta \end{array}$$

### Appendix A.3. The Eigenvalues and Eigenvectors of the Disease-Free Equilibrium point

The eigenvectors corresponding to the eigenvalues for disease-free equilibrium points are as follows:

(A9) $$\begin{array}{cccc} \lambda_{1} & =0,\; & v_{1} & =\left[-\frac{\gamma C_{M}}{\alpha^{\left(B\right)}},0,0,0,0,0,1\right] \end{array}$$

(A10) $$\begin{array}{cccc} \lambda_{2} & =-\frac{\gamma \left({f_{I}}^{\left(wt\right)}\alpha^{\left(B\right)}-\alpha^{\left(wt\right)}\right)}{\alpha^{\left(B\right)}},\; & v_{2} & =\left[v_{21},v_{22},0,1,v_{25},0,v_{27}\right] \end{array}$$

(A11) $$\begin{array}{cccc} \lambda_{3} & =-\frac{\gamma \left({f_{I}}^{\left(mut\right)}\alpha^{\left(B\right)}-\alpha^{\left(mut\right)}\right)}{\alpha^{\left(B\right)}},\; & v_{3} & =\left[v_{31},0,v_{33},1,0,v_{36},v_{37}\right] \end{array}$$

(A12) $$\begin{array}{cccc} \lambda_{4} & =-\frac{\beta \;\left(\alpha^{\left(B\right)}-\alpha^{\left(wt\right)}\right)}{\alpha^{\left(B\right)}},\; & v_{4} & =\left[0,0,0,1,-\frac{{\left(\alpha^{\left(B\right)}\right)}^{2}-2\alpha^{\left(B\right)}\beta +\beta \alpha^{\left(wt\right)}}{\alpha^{\left(B\right)}\left(\alpha^{\left(B\right)}-\beta \right)},0,0\right] \end{array}$$

(A13) $$\begin{array}{cccc} \lambda_{5} & =-\frac{\beta \left(\alpha^{\left(B\right)}-\alpha^{\left(\mathrm{mut}\right)}\right)}{\alpha^{\left(B\right)}},\; & v_{5} & =\left[0,0,0,1,0,-\frac{{\left(\alpha^{\left(B\right)}\right)}^{2}-2\alpha^{\left(B\right)}\beta +\beta \alpha^{\left(mut\right)}}{\alpha^{\left(B\right)}\left(\alpha^{\left(B\right)}-\beta \right)},0\right] \end{array}$$

(A14) $$\begin{array}{cccc} \lambda_{6} & =-\alpha^{\left(B\right)}+\beta ,\; & v_{6} & =\left[0,0,0,1,0,0,0\right] \end{array}$$

(A15) $$\begin{array}{cccc} \lambda_{7} & =-\alpha^{\left(B\right)}+\gamma ,\; & v_{7} & =\left[1,0,0,0,0,0,0\right] \end{array}$$

### Appendix A.4. The Eigenvalues wt Strain Equilibrium

The eigenvalues of the equilibrium of wt strain are calculated as

(A16) $$\begin{array}{cc} \lambda_{1} & =-C_{I}\gamma \left({f_{I}}^{\left(mut\right)}-\frac{\alpha^{\left(mut\right)}{f_{I}}^{\left(wt\right)}}{\alpha^{\left(wt\right)}}\right) \end{array}$$

(A17) $$\begin{array}{cc} \lambda_{2} & =-\alpha^{\left(wt\right)}\left(1-\frac{C_{I}\gamma \;{f_{I}}^{\left(wt\right)}}{\alpha^{\left(wt\right)}}\right) \end{array}$$

(A18) $$\begin{array}{cc} \lambda_{3} & =-C_{I}\gamma \left(1-\frac{\alpha^{\left(B\right)}{f_{I}}^{\left(wt\right)}}{\alpha^{\left(wt\right)}}\right) \end{array}$$

(A19) $$\begin{array}{cc} \lambda_{4} & =-\frac{\kappa \;C_{M}}{C_{I}}\left(1-\frac{C_{I}\gamma \;{f_{I}}^{\left(wt\right)}}{\alpha^{\left(wt\right)}}\right) \end{array}$$

(A20) $$\begin{array}{cc} \lambda_{5} & =-\alpha^{\left(wt\right)}\sqrt{4\frac{C_{M}}{C_{L}}{\left(1-\frac{C_{I}\gamma f_{I}^{\left(wt\right)}}{\alpha^{\left(wt\right)}}\right)}^{2}+{\left(1-\frac{\beta }{\alpha^{\left(wt\right)}}\right)}^{2}} \end{array}$$

(A21) $$\begin{array}{cc} \lambda_{6} & =-\frac{1}{2}\left(2\beta -\alpha^{\left(B\right)}\left(1+\frac{\beta }{\alpha^{\left(wt\right)}}\right)+\alpha^{\left(B\right)}\sqrt{4\frac{C_{M}}{C_{L}}{\left(1-\frac{C_{I}\gamma f_{I}^{\left(wt\right)}}{\alpha^{\left(wt\right)}}\right)}^{2}+{\left(1-\frac{\beta }{\alpha^{\left(wt\right)}}\right)}^{2}}\right) \end{array}$$

(A22) $$\begin{array}{cc} \lambda_{7} & =-\frac{1}{2}\left(2\beta -\alpha^{\left(mut\right)}\left(1+\frac{\beta }{\alpha^{\left(wt\right)}}\right)+\alpha^{\left(mut\right)}\sqrt{4\frac{C_{M}}{C_{L}}{\left(1-\frac{C_{I}\gamma f_{I}^{\left(wt\right)}}{\alpha^{\left(wt\right)}}\right)}^{2}+{\left(1-\frac{\beta }{\alpha^{\left(wt\right)}}\right)}^{2}}\right) \end{array}$$

### Appendix A.5. The Eigenvalues mut Strain Equilibrium

The eigenvalues of equilibrium of mut strain are calculated as

(A23) $$\begin{array}{cc} \lambda_{1} & =-C_{I}\gamma \left({f_{I}}^{\left(wt\right)}-\frac{\alpha^{\left(wt\right)}{f_{I}}^{\left(mut\right)}}{\alpha^{\left(mut\right)}}\right) \end{array}$$

(A24) $$\begin{array}{cc} \lambda_{2} & =-\alpha^{\left(mut\right)}\left(1-\frac{C_{I}\gamma \;{f_{I}}^{\left(mut\right)}}{\alpha^{\left(mut\right)}}\right) \end{array}$$

(A25) $$\begin{array}{cc} \lambda_{3} & =-C_{I}\gamma \left(1-\frac{\alpha^{\left(B\right)}{f_{I}}^{\left(mut\right)}}{\alpha^{\left(mut\right)}}\right) \end{array}$$

(A26) $$\begin{array}{cc} \lambda_{4} & =-\frac{\kappa \;C_{M}}{C_{I}}\left(1-\frac{C_{I}\gamma \;{f_{I}}^{\left(mut\right)}}{\alpha^{\left(mut\right)}}\right) \end{array}$$

(A27) $$\begin{array}{cc} \lambda_{5} & =-\alpha^{\left(mut\right)}\sqrt{4\frac{C_{M}}{C_{L}}{\left(1-\frac{C_{I}\gamma f_{I}^{\left(mut\right)}}{\alpha^{\left(mut\right)}}\right)}^{2}+{\left(1-\frac{\beta }{\alpha^{\left(mut\right)}}\right)}^{2}} \end{array}$$

(A28) $$\begin{array}{cc} \lambda_{6} & =-\frac{1}{2}\left(2\beta -\alpha^{\left(B\right)}\left(1+\frac{\beta }{\alpha^{\left(mut\right)}}\right)+\alpha^{\left(B\right)}\sqrt{4\frac{C_{M}}{C_{L}}{\left(1-\frac{C_{I}\gamma f_{I}^{\left(mut\right)}}{\alpha^{\left(mut\right)}}\right)}^{2}+{\left(1-\frac{\beta }{\alpha^{\left(mut\right)}}\right)}^{2}}\right) \end{array}$$

(A29) $$\begin{array}{cc} \lambda_{7} & =-\frac{1}{2}\left(2\beta -\alpha^{\left(wt\right)}\left(1+\frac{\beta }{\alpha^{\left(mut\right)}}\right)+\alpha^{\left(wt\right)}\sqrt{4\frac{C_{M}}{C_{L}}{\left(1-\frac{C_{I}\gamma f_{I}^{\left(mut\right)}}{\alpha^{\left(mut\right)}}\right)}^{2}+{\left(1-\frac{\beta }{\alpha^{\left(mut\right)}}\right)}^{2}}\right) \end{array}$$

### Appendix A.6. The Basic Reproduction Numbers

The basic reproduction number is calculated as follows:

1. Based on Equations (14) and (15), we split (22)–(28) into two compartments: one disease compartment with populations $\left(Y_{M}^{\left(wt\right)},Y_{M}^{\left(mut\right)},Y_{L}^{\left(wt\right)},Y_{L}^{\left(mut\right)}\right)$ and one non-disease compartment with populations $\left(B_{M},B_{L},I\right)$.
2. We consider the first four Equations (22)–(28) correspond to the appearance of a new infection as well as the rate of other transitions between other infected compartments.
3. $F_{i}\left(x\right)$ corresponds to the rate of the appearance of new infections, and $V_{i}$ corresponds to the rate of transfer assumed as
  (A30) $$\begin{array}{cc} F_{1} & =\left(\alpha^{\left(wt\right)}-\sigma_{M⟶L}^{\left(wt\right)}\right)\;\cdot \;Y_{M}^{\left(wt\right)} \end{array}$$
  (A31) $$\begin{array}{cc} F_{2} & =\left(\alpha^{\left(mut\right)}-\sigma_{M⟶L}^{\left(mut\right)}\right)\;\cdot \;Y_{M}^{\left(mut\right)} \end{array}$$
  (A32) $$\begin{array}{cc} F_{3} & =\left(\alpha_{L}^{\left(wt\right)}\right)\;\cdot \;Y_{L}^{\left(wt\right)} \end{array}$$
  (A33) $$\begin{array}{cc} F_{4} & =\left(\alpha_{L}^{\left(mut\right)}\right)\;\cdot \;Y_{L}^{\left(mut\right)} \end{array}$$
  and
  (A34) $$\begin{array}{cc} V_{1} & =\left(\gamma \;\cdot \;f_{I}^{\left(wt\right)}\;\cdot \;I\right)\;\cdot \;Y_{M}^{\left(wt\right)} \end{array}$$
  (A35) $$\begin{array}{cc} V_{2} & =\left(\gamma \;\cdot \;f_{I}^{\left(mut\right)}\;\cdot \;I\right)\;\cdot \;Y_{M}^{\left(mut\right)} \end{array}$$
  (A36) $$\begin{array}{cc} V_{3} & =\left(\beta \right)\;\cdot \;Y_{L}^{\left(wt\right)}-\sigma_{M⟶L}^{\left(wt\right)}\;\cdot \;Y_{M}^{\left(wt\right)} \end{array}$$
  (A37) $$\begin{array}{cc} V_{4} & =\left(\beta \right)\;\cdot \;Y_{L}^{\left(mut\right)}-\sigma_{M⟶L}^{\left(mut\right)}\;\cdot \;Y_{M}^{\left(mut\right)} \end{array}$$
4. The basic reproduction number is achieved as the spectral radius of the matrix $\left(FV^{-1}\right)$;
  as $R=\rho \left(FV^{-1}\right)$ where $F=\frac{\partial F_{i}\left(P^{0}\right)}{\partial x_{j}}$, $V=\frac{\partial V_{i}\left(P^{0}\right)}{\partial x_{j}}$, and $x_{j}$ are as follows:
  (A38) $$\begin{array}{c} x_{j}=\left(Y_{M}^{\left(wt\right)},Y_{M}^{\left(mut\right)},Y_{L}^{\left(wt\right)},Y_{L}^{\left(mut\right)}\right) \end{array}$$
  (A39) $$\begin{array}{c} F=\left(\begin{array}{cccc} \alpha^{\left(wt\right)}(\frac{\gamma }{\alpha^{\left(B\right)})} & 0 & 0 & 0\\ 0 & \alpha^{\left(mut\right)}(\frac{\gamma }{\alpha^{\left(B\right)})} & 0 & 0\\ 0 & 0 & \alpha^{\left(wt\right)}(\frac{\beta }{\alpha^{\left(B\right)})} & 0\\ 0 & 0 & 0 & \alpha^{\left(mut\right)}(\frac{\beta }{\alpha^{\left(B\right)})} \end{array}\right) \end{array}$$
  and
  (A40) $$\begin{array}{c} V=\left(\begin{array}{cccc} \gamma \;{f_{I}}^{\left(wt\right)} & 0 & 0 & 0\\ 0 & \gamma \;{f_{I}}^{\left(mut\right)} & 0 & 0\\ -\frac{\alpha^{\left(wt\right)}\left(-\gamma +\alpha^{\left(B\right)}\right)}{\alpha^{\left(B\right)}} & 0 & \beta & 0\\ 0 & -\frac{\alpha^{\left(mut\right)}\left(-\gamma +\alpha^{\left(B\right)}\right)}{\alpha^{\left(B\right)}} & 0 & \beta \end{array}\right). \end{array}$$
  Then
  (A41) $$\begin{array}{cc} R & =\rho \left(FV^{-1}\right) \end{array}$$
  (A42) $$\begin{array}{cc} \; & =\max\left\{\left|\frac{\alpha^{\left(wt\right)}}{\alpha^{\left(B\right)}}\right|,\left|\frac{\alpha^{\left(mut\right)}}{\alpha^{\left(B\right)}}\right|,\left|\frac{\alpha^{\left(wt\right)}}{\alpha^{\left(B\right)}{f_{I}}^{\left(wt\right)}}\right|,\left|\frac{\alpha^{\left(mut\right)}}{\alpha^{\left(B\right)}{f_{I}}^{\left(mut\right)}}\right|\right\} \end{array}$$

## Appendix B. Data Availability

1. The Matlab script
2. The Maple workbook
3. The described model is available in SBML format [26] (Level 3 Version 2 [27]) from BioModels database [36] under model identifier MODEL2002070001.
